# Cardiac sympathetic burden reflects Parkinson disease burden, regardless of high or low orthostatic blood pressure changes

**DOI:** 10.1038/s41531-021-00217-3

**Published:** 2021-08-12

**Authors:** Sang-Won Yoo, Joong-Seok Kim, Yoon-Sang Oh, Dong-Woo Ryu, Seunggyun Ha, Ji-Yeon Yoo, Kwang-Soo Lee

**Affiliations:** 1 Department of Neurology, College of Medicine, The Catholic University of Korea, Seoul, Republic of Korea; 2 Division of Nuclear Medicine, Department of Radiology, College of Medicine, The Catholic University of Korea, Seoul, Republic of Korea

**Keywords:** Parkinson's disease, Parkinson's disease

## Abstract

Reduced uptake of ^123^I-*meta*-iodobenzylguanidine (^123^I-MIBG) and orthostatic hypotension (OH) are independently associated with worse clinical outcomes of Parkinson’s disease (PD). However, their interactive influence on PD has not been studied. The role of ^123^I-MIBG myocardial uptake, as a biomarker of PD severity, was investigated, conditional on the mediating effects of OH. A total of 227 PD patients were enrolled. Their motor and nonmotor aspects were assessed with standardized tools. Global disease burden was estimated by averaging the scaled z-scores of the assessment tools. Every patient went through ^123^I-MIBG scan, and OH was evaluated with the head-up tilt-test. The mediating role of orthostatic blood pressure changes (ΔBP) on the association between cardiac sympathetic denervation and disease burden was investigated. Low heart-to-mediastinum (H/M) ratio with less than 1.78 was seen in 69.6% of the patient population, and 22.9% of patients had OH. Low H/M ratio was associated with OH, and these patients had worse disease burden than subjects with normal ^123^I-MIBG uptake (global composite z-score: normal ^123^I-MIBG vs. abnormal ^123^I-MIBG; −0.3 ± 0.5 vs. 0.1 ± 0.7; *p* < 0.001). The mediation models, controlled for age and disease duration, revealed that the delayed H/M ratio and global composite score were negatively associated, irrespective of orthostatic ΔBP. Adverse relationship between cardiac sympathetic denervation and disease burden was shown without any interference from orthostatic blood pressure fluctuations. This result suggested that extracranial cardiac markers might reflect disease burden, regardless of labile blood pressure influence.

## Introduction

Cardiovascular dysautonomia is increasingly accepted as a prodromal ‘window’ in which Parkinson’s disease (PD) can be detected^1–5^. These findings have been reported as clinical biomarkers for predicting PD clinical outcomes and consequences^6–10^.

Cardiac postganglionic sympathetic denervation, reflected more accurately in the decreased delayed heart-to-mediastinum (H/M) ratio upon ^123^I-*meta*-iodobenzylguanidine (^123^I-MIBG) myocardial scintigraphy scan^11^, contributes to orthostatic hypotension (OH)^4^. Reduced myocardial uptake of ^123^I-MIBG indicates norepinephrine transporter dysfunction in sympathetic neurons, and may reflect physiological consequences in residual functional cardiac sympathetic axons^7,12^. Reduced uptake of ^123^I-MIBG was utilized to discriminate PD from vascular Parkinsonism, drug-induced Parkinsonism, and atypical parkinsonian syndromes (APS)^2^.

Some PD patients have a normal ^123^I-MIBG uptake analogous to APS, and this imaging phenotype was previously given the term “scans without evidence of cardiac norepinephrine deficit (SWEND)”^13^. PD patients with SWEND had mild Hoehn and Yahr (H&Y) stage, short disease duration, slow progression of motor dysfunction, a lower incidence of the wearing-off phenomenon, and a lower prevalence of nonmotor symptoms^7,10–12,14,15^.

Most studies have investigated the association of cardiac sympathetic denervation with orthostatic hypotension and the impact on clinical outcomes separately, but the interacting effects of these two biomarkers on PD patient symptoms have seldom been explored^4,16^. As a pathophysiologic contributor, OH is a possible biomarker of pathologic burdens^2,4,7,12,17^, thus the confounding effects of OH need to be considered when assessing the role of ^123^I-MIBG myocardial scintigraphy results in PD patients.

In this study, we investigated whether a mediating role for OH could be disproved in the context of a significant association between cardiac sympathetic denervation and clinical disease burden. The refutation of this role would emphasize the sustained value of ^123^I-MIBG uptakes as a pathologic biomarker of disease severity despite its contribution to OH.

Orthostatic hypertension (OHT) is also a type of cardiovascular dysregulation which can be anticipated in PD patients^18,19^. This study also aimed to document the nature of OHT in early PD patients.

## Results

### Baseline characteristics

Baseline characteristics are summarized in Table 1. A total of 227 patients with mild PD stage were included. The mean age of the population was 69.6 ± 9.2 years old, and 105 (46.3%) were female. The disease duration was 1.1 ± 1.0 years. Total Unified Parkinson’s Disease Rating Scale (UPDRS) and H&Y were 23.4 ± 11.9 and 2.0 (Interquartile range, IQR, 0.0), respectively. The Mini-Mental Status Examination (MMSE) was 26.8 ± 3.0 and Clinical Dementia Rating (CDR) was 0.5 (IQR, 0.0). The mean uptake of early and delayed ^123^I-MIBG H/M ratio was 1.58 ± 0.31 and 1.55 ± 0.37, respectively. 69.6% (158/227) of patients were defined as having decreased H/M ratio on ^123^I-MIBG scintigraphy (normal ^123^I-MIBG group vs. abnormal ^123^I-MIBG group: 2.04 ± 0.16 vs. 1.34 ± 0.18, respectively). Twenty patients were found to be hypertensive when in the supine position; 40% (8/20) and 30% (6/20) of patients with supine hypertension (SH) had co-existing OH and OHT, respectively. Among the patients studied, 22.9% (52/227) and 26.4% (60/227) were classified into OH and OHT groups, respectively.

**Table 1:** Baseline characteristics.

	PD (*n* = 227)	Normal ^123^I-MIBG (*n* = 69)	Abnormal ^123^I-MIBG (*n* = 158)	*p* value
Age, years, mean (SD)	69.6 (9.2)	68.9 (9.9)	69.9 (8.8)	0.445
Sex, female, *n* (%)	105 (46.3)	35 (50.7)	70 (44.3)	0.389
Body mass index, kg/m^2^, mean (SD)	23.7 (3.1)	23.4 (3.0)	23.9 (3.1)	0.310
Disease duration, years, mean (SD)	1.1 (1.0)	1.0 (1.0)	1.2 (1.0)	0.399
Diabetes mellitus, *n* (%)	39 (17.2)	14 (20.3)	25 (15.8)	0.446
Dyslipidemia, *n* (%)	62 (27.3)	21 (30.4)	41 (25.9)	0.519
Hypertension, *n* (%)	95 (41.9)	27 (39.1)	68 (43.0)	0.661
Non-smoker, *n* (%)	222 (97.8)	68 (98.6)	154 (97.5)	1.000
UPDRS, total, mean (SD)	23.4 (11.9)	20.4 (11.1)	24.8 (12.0)	0.010
UPDRS Part I, mean (SD)	1.6 (1.4)	1.4 (1.3)	1.7 (1.4)	0.222
UPDRS Part II, mean (SD)	6.1 (4.1)	5.3 (4.0)	6.4 (4.1)	0.074
UPDRS Part III, mean (SD)	15.8 (8.6)	13.6 (8.0)	16.7 (8.7)	0.013
H&Y stage, median (IQR)	2.0 (0.0)	2.0 (1.0)	2.0 (0.0)	0.112
H&Y stage^a^				0.307
H&Y stage 1, *n* (%)	55 (24.2)	22 (31.9)	33 (20.9)	
H&Y stage 2, *n* (%)	146 (64.3)	40 (58.0)	106 (67.1)	
H&Y stage 3, *n* (%)	25 (11.0)	7 (10.1)	18 (11.4)	
H&Y stage 4, *n* (%)	1 (0.4)	0 (0.0)	1 (0.6)	
MMSE, mean (SD)	26.8 (3.0)	26.8 (3.4)	26.9 (2.9)	0.844
CDR, median (IQR)	0.5 (0.0)	0.5 (0.0)	0.5 (0.0)	0.812
Early H/M ratio, mean (SD)	1.58 (0.31)	1.96 (0.18)	1.41 (0.18)	<0.001
Delayed H/M ratio, mean (SD)	1.55 (0.37)	2.04 (0.16)	1.34 (0.18)	<0.001
Washout rate, mean (SD)	2.18 (8.37)	−4.87 (6.81)	5.25 (7.03)	<0.001
Supine SBP, mean (SD)	124.1 (16.5)	122.7 (17.2)	124.6 (16.2)	0.428
Supine DBP, mean (SD)	71.0 (9.1)	70.1 (9.0)	71.4 (9.1)	0.317
orthostatic ΔSBP_min_, mean (SD)	10.6 (13.3)	5.3 (11.0)	12.9 (13.6)	<0.001
orthostatic ΔDBP_min_, mean (SD)	2.8 (7.8)	0.4 (6.8)	3.8 (8.0)	0.002
orthostatic ΔSBP_max_, mean (SD)	0.9 (13.5)	−3.3 (11.8)	2.7 (13.8)	0.002
orthostatic ΔDBP_max_, mean (SD)	−4.3 (7.6)	−5.9 (6.9)	−3.5 (7.8)	0.030
SH, *n* (%)	20 (8.8)	8 (11.6)	12 (7.6)	0.321
OH, *n* (%)	52 (22.9)	7 (10.1)	45 (28.5)	0.002
OHT, *n* (%)	60 (26.4)	26 (37.7)	34 (21.5)	0.014
NMSS, total, median (IQR)	18.0 (30.0)	15.0 (23.0)	19.5 (33.5)	0.010
MADRS, total, median (IQR)	4.0 (7.0)	3.0 (5.0)	4.0 (7.0)	0.024
ESS, total, median (IQR)	2.0 (3.5)	2.0 (3.0)	3.0 (3.0)	0.012
PDSS2, total, mean (SD)	7.4 (6.8)	5.3 (5.2)	8.3 (7.2)	0.001
RBDSQ, total, mean (SD)	3.2 (2.6)	1.9 (1.5)	3.8 (2.7)	<0.001
SCOPA-AUT, total, mean (SD)	8.1 (6.7)	6.2 (5.7)	8.9 (6.9)	0.003
OHQ Part I, subtotal, median (IQR)	0.0 (9.0)	0.0 (4.0)	0.0 (10.0)	0.133
OHQ Part II, subtotal, median (IQR)	0.0 (7.0)	0.0 (2.0)	0.0 (8.0)	0.004
Global composite score, z, mean (SD)	0.0 (0.7)	−0.3 (0.5)	0.1 (0.7)	<0.001
Motor composite score, z, mean (SD)	0.0 (0.89)	−0.2 (0.9)	0.1 (0.9)	0.016
Sleep composite score, z, mean (SD)	0.0 (0.76)	−0.4 (0.5)	0.2 (0.8)	<0.001
Autonomic composite score, z, mean (SD)	0.0 (0.80)	−0.2 (0.7)	0.1 (0.8)	0.006

The values of blood pressures are expressed in mmHg.

Independent *t*-test or Welch’s *t*-test was conducted for continuous variables and Fisher’s exact test was used for categorical variables. Non-normally distributed variables were analyzed by Mann–Whitney *U* tests.

*PD* Parkinson’s disease, *MIBG* metaiodobenzylguanidine, *UPDRS* Unified Parkinson’s Disease Rating Scale, *H&Y* Hoehn and Yahr, *MMSE* Mini-Mental Status Examination, *CDR* Clinical Dementia Rating, *SBP* systolic blood pressure, *DBP* diastolic blood pressure, *SH* supine hypertension, *OH* orthostatic hypotension, *OHT* orthostatic hypertension, *H/M* heart-to-mediastinum, *NMSS* Nonmotor Symptoms Scale, *MADRS* Montgomery-Asberg Depression Rating Scale, *ESS* Epworth Sleepiness Scale, *PDSS2* Parkinson’s Disease Sleep Scale-2, *RBDSQ* REM Sleep Behavior Disorder Screening Questionnaire, *SCOPA-AUT* Scale for Outcomes in Parkinson’s Disease-Autonomic, *OHQ* Orthostatic Hypotension Questionnaire.

^a^Fisher’s exact test was performed between H&Y stages and MIBG groups.

### Comparisons between normal ^123^I-MIBG group and abnormal ^123^I-MIBG group

The group with low delayed H/M ratio had worse outcomes on motor and nonmotor measurements. Total and Part III UPDRS scores were higher (normal ^123^I-MIBG group vs. abnormal ^123^I-MIBG group: total UPDRS, 20.4 ± 11.1 vs. 24.8 ± 12.0, respectively, *p* = 0.010; and for Part III, 13.6 ± 8.0 vs. 16.7 ± 8.7, respectively, *p* = 0.013). H&Y stages did not differ between groups (normal ^123^I-MIBG group vs. abnormal ^123^I-MIBG group: 2.0 [1.0] vs. 2.0 [0.0], *p* = 0.112; Fisher’s exact test, *p* = 0.307). The scores of Nonmotor Symptoms Scale (NMSS), Montgomery-Asberg Depression Rating Scale (MADRS), Epworth Sleepiness Scale (ESS), Parkinson’s Disease Sleep Scale-2 (PDSS-2), REM Sleep Behavior Disorder Screening Questionnaire (RBDSQ), Scale for Outcomes in Parkinson’s Disease-Autonomic (SCOPA-AUT), and the Orthostatic Hypotension Questionnaire (OHQ) Part II were compared between groups; the abnormal ^123^I-MIBG group was scored higher than the normal ^123^I-MIBG group. PD patients with decreased delayed H/M ratio had worse overall disease burden than PD patients with normal ^123^I-MIBG (normal ^123^I-MIBG group vs. abnormal ^123^I-MIBG group: global composite score, −0.3 ± 0.5 vs. 0.1 ± 0.7, respectively, *p* < 0.001).

Decreased ^123^I-MIBG uptake was associated with OH, while normal ^123^I-MIBG uptake was associated with OHT (Fisher’s exact test, *p* = 0.002, *p* = 0.014; respectively). The magnitude of blood pressure (BP) drop was higher in the abnormal ^123^I-MIBG group (normal ^123^I-MIBG vs. abnormal ^123^I-MIBG: ΔSBP_min_, 5.3 ± 11.0 vs. 12.9 ± 13.6, respectively, *p* < 0.001; ΔDBP_min_, 0.4 ± 6.8 vs. 3.8 ± 8.0, respectively, *p* = 0.002), and BP rise was greater in the normal ^123^I-MIBG group (normal ^123^I-MIBG vs. abnormal ^123^I-MIBG: ΔSBP_max_, −3.3 ± 11.8 vs. 2.7 ± 13.8, respectively, *p* = 0.002; ΔDBP_max_, −5.9 ± 6.9 vs. −3.5 ± 7.8, respectively, *p* = 0.030).

### The mediating effects of orthostatic blood pressure changes on clinical outcomes

**Fig. 1:**
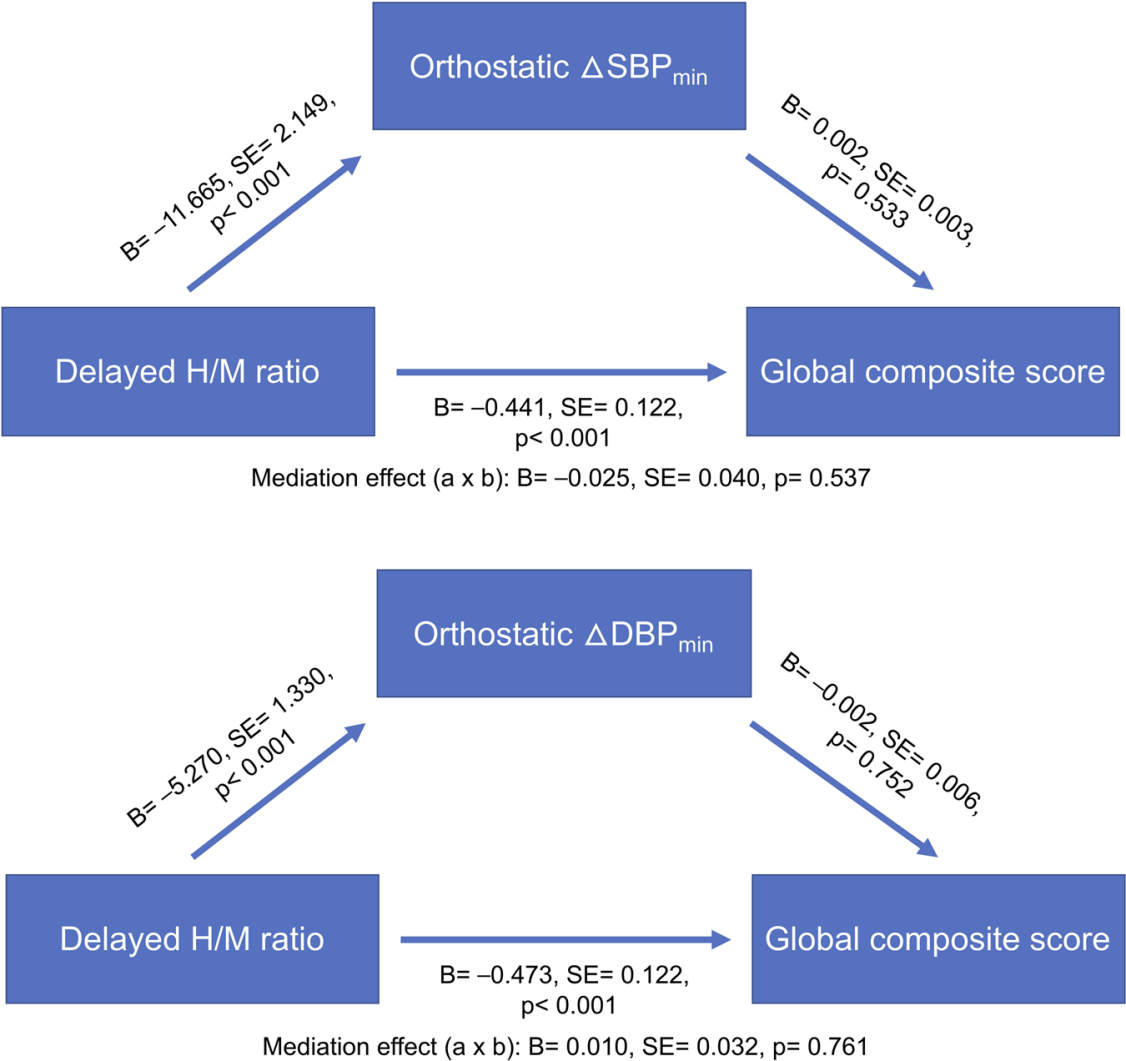
Path diagram of mediation analysis for global composite score. *H/M* heart-to-mediastinum, *SBP* systolic blood pressure, *DBP* diastolic blood pressure, *B* estimate, *SE* standard error.

Table 2 and Fig. 1 present the mediating effects of BP changes (∆SBP_min_, ∆DBP_min_) on global composite score. When controlling for age and disease duration, delayed H/M ratio was negatively associated with global composite score (*total* effect [*indirect* + *direct*]: CI, [−0.701, −0.226]). Delayed H/M ratio was also negatively associated with orthostatic ∆BP_min_ (component ***a*** → ***b***: ΔSBP_min_ vs. ΔDBP_min_; CI, [−15.987, −7.355] vs. CI, [−7.966, −2.530]), but its inverse association was not maintained (component ***b*** → ***c***: ΔSBP_min_ vs. ΔDBP_min_; CI, [−0.004, 0.009] vs. CI, [−0.013, 0.009]). The overall *indirect* influence was not significant (***a*** → ***b*** → ***c***: ΔSBP_min_ vs. ΔDBP_min_; CI, [−0.106, 0.051] vs. CI, [−0.049, 0.075]), but the *direct* negative associations between delayed H/M ratio and global composite score were maintained (component ***a*** → ***c***: ΔSBP_min_ vs. ΔDBP_min_; CI, [−0.687, −0.200] vs. CI, [−0.710, −0.237]). The results were similar when early H/M ratio (***a’***) and washout rate (***a”***) were set as predictors in the mediation analyses (Supplementary Table 1, Supplementary Fig. 1).

**Table 2:** The mediating effects of orthostatic blood pressure changes (∆SBP_min_, ∆DBP_min_) on global composite score.

Predictor (a): delayed H/M ratio								
Mediator (b): ΔSBP_min_								
Type	Effect	Estimate	SE	Lower 95% CI	Upper 95% CI	β	*z*	*p*
Indirect	a → b → c	−0.025	0.040	−0.106	0.051	−0.013	−0.617	0.537
Component	a → b	−11.665	2.149	−15.987	−7.355	−0.325	−5.428	<0.001
	b → c	0.002	0.003	−0.004	0.009	0.040	0.624	0.533
Direct	a → c	−0.441	0.122	−0.687	−0.200	−0.231	−3.620	<0.001
Total	Indirect + direct	−0.464	0.121	−0.701	−0.226	−0.242	−3.823	<0.001
Mediator (b): ΔDBP_min_								
Type	Effect	Estimate	SE	Lower 95% CI	Upper 95% CI	β	*z*	*p*
Indirect	a → b → c	0.010	0.032	−0.049	0.075	0.005	0.304	0.761
Component	a → b	−5.270	1.330	−7.966	−2.530	−0.251	−3.962	<0.001
	b → c	−0.002	0.006	−0.013	0.009	−0.020	−0.316	0.752
Direct	a → c	−0.473	0.122	−0.710	−0.237	−0.247	−3.862	<0.001
Total	Indirect + direct	−0.464	0.121	−0.701	−0.226	−0.242	−3.823	<0.001

Confidence intervals (CIs) were calculated with percentile bootstrap (*n* = 1000) method. Betas are completely standardized effect sizes

The mediation model was controlled by age and disease duration. Multicollinearity was prevented by mean centering method.

*SBP* systolic blood pressure, *DBP* diastolic blood pressure, *SE* standard error, *a* Delayed heart-to-mediastinum (H/M) ratio, *b* ∆SBP_min_ or ∆DBP_min_, *c* global composite score.

The subdomains of global composite score, partitioned by motor, sleep, and autonomic composite scores were analyzed (Table 3). A delayed H/M ratio negatively predicted each domain (*total* effect), and its inverse influence was not mediated by orthostatic ΔBP (*indirect* effect).

**Table 3:** The mediating effects of orthostatic blood pressure changes (∆SBP_min_, ∆DBP_min_) on subdomain composite scores.

Predictor (a): delayed H/M ratio								
Motor composite score (c’)								
Mediator (b): ΔSBP_min_								
Type	Effect	Estimate	SE	Lower 95% CI	Upper 95% CI	β	*z*	*p*
Indirect	a → b → c’	−0.069	0.069	−0.219	0.058	−0.029	−0.100	0.319
Component	a → b	−11.665	2.214	−15.711	−6.766	−0.325	−5.268	<0.001
	b → c’	0.006	0.006	−0.005	0.017	0.089	1.046	0.295
Direct	a → c’	−0.300	0.146	−0.589	−0.015	−0.126	−2.055	0.040
Total	Indirect + direct	−0.363	0.146	−0.650	−0.077	−0.151	−2.485	0.013
Mediator (b): ΔDBP_min_								
Type	Effect	Estimate	SE	Lower	Upper	β	*z*	*p*
Indirect	a → b → c’	−0.033	0.048	−0.139	0.054	−0.014	−0.688	0.492
Component	a → b	−5.270	1.361	−7.870	−2.670	−0.251	−3.873	<0.001
	b → c’	0.006	0.009	−0.011	0.025	0.055	0.707	0.479
Direct	a → c’	−0.334	0.147	−0.621	−0.037	−0.139	−2.269	0.023
Total	Indirect + direct	−0.363	0.146	−0.650	−0.077	−0.151	−2.485	0.013
Sleep composite score(c”)								
Mediator (b): ΔSBP_min_								
Type	Effect	Estimate	SE	Lower 95% CI	Upper 95% CI	β	*z*	*p*
Indirect	a → b → c”	−0.061	0.046	−0.161	0.026	−0.030	−1.333	0.183
Component	a → b	−11.665	2.207	−16.194	−7.582	−0.325	−5.285	<0.001
	b → c”	0.005	0.004	−0.002	0.013	0.091	1.415	0.157
Direct	a → c”	−0.613	0.124	−0.850	−0.370	−0.298	−4.960	<0.001
Total	Indirect + direct	−0.669	0.130	−0.923	−0.415	−0.325	−5.163	<0.001
Mediator (b): ΔDBP_min_								
Type	Effect	Estimate	SE	Lower 95% CI	Upper 95% CI	β	*z*	*p*
Indirect	a → b → c”	−0.005	0.032	−0.072	0.063	−0.003	−0.161	0.872
Component	a → b	−5.270	1.316	−7.997	−2.770	−0.251	−4.006	<0.001
	b → c”	0.001	0.006	−0.011	0.014	0.010	0.166	0.868
Direct	a → c”	−0.665	0.133	−0.924	−0.409	−0.323	−5.009	<0.001
Total	Indirect + direct	−0.669	0.130	−0.923	−0.415	−0.325	−5.163	<0.001
Autonomic composite score(c”’)								
Mediator (b): ΔSBP_min_								
Type	Effect	Estimate	SE	Lower 95% CI	Upper 95% CI	β	*z*	*p*
Indirect	a → b → c”’	0.010	0.043	−0.073	0.097	0.005	0.224	0.822
Component	a → b	−11.665	2.110	−15.643	−7.259	−0.325	−5.527	<0.001
	b → c”’	−0.153	0.004	−0.008	0.006	−0.014	−0.226	0.821
Direct	a → c”’	−0.356	0.144	−0.650	−0.073	−0.165	−2.476	0.013
Total	Indirect + direct	−0.347	0.141	−0.624	−0.071	−0.161	−2.463	0.014
Mediator (b): ΔDBP_min_								
Type	Effect	Estimate	SE	Lower 95% CI	Upper 95% CI	β	*z*	*p*
Indirect	a → b → c”’	0.018	0.034	−0.041	0.097	0.008	0.532	0.594
Component	a → b	−5.270	1.317	−7.836	−2.771	−0.251	−4.000	<0.001
	b → c”’	−0.003	0.006	−0.016	0.009	−0.034	−0.565	0.572
Direct	a → c”’	−0.364	0.137	−0.629	−0.091	−0.168	−2.650	0.008
Total	Indirect + direct	−0.347	0.141	−0.624	−0.071	−0.161	−2.463	0.014

Confidence intervals (CIs) were calculated with percentile bootstrap (*n* = 1000) method. Betas are completely standardized effect sizes.

The mediation model was controlled by age and disease duration. Multicollinearity was prevented by mean centering method.

*SBP* systolic blood pressure, *DBP* diastolic blood pressure, *SE* standard error, *a* Delayed heart-to-mediastinum (H/M) ratio, *b* ∆SBP_min_ or ∆DBP_min_, *c’* motor composite score, *c”* sleep composite score, *c”’* autonomic composite score.

### Bidirectionality of orthostatic blood pressure change

In an observational sub-analysis of orthostatic ∆BP_max_, OHT group displayed a worsening trend for global composite score as the ∆BP_max_ decreased while the non-OHT group demonstrated a positive association as the ΔBP_max_ increased, especially ΔSBP_max_ (Supplementary Fig. 2). These associations lacked any statistical significance; however, orthostatic BP rise and drop was accompanied by trends toward adverse disease severity in both directions.

### Comparison of washout rate between OHT and non-OHT

The washout rate (WR) was significantly opposite (normal ^123^I-MIBG vs. abnormal ^123^I-MIBG: WR, −4.87 ± 6.81 vs. 5.25 ± 7.03, respectively, *p* < 0.001). In a sub-analysis of sixty-nine normal ^123^I-MIBG patients, 37.7% (26/69) were orthostatic hypertensive. The WR was compared between those with OHT and without OHT. The comparison did not reveal any significant difference (non-OHT vs. OHT: −4.39 ± 7.29 vs. −5.65 ± 5.99, respectively; independent *t*-test, *t* = 0.743, *df* = 67.0, *p* = 0.460).

## Discussion

Cardiac sympathetic denervation and orthostatic hypotension were encountered in early PD patients. OH was found more frequently in the abnormal ^123^I-MIBG group while orthostatic hypertension was more common in SWEND PD patients. PD patients with reduced ^123^I-MIBG uptake presented with worse clinical features. Cardiac denervation inversely predicted PD-related disease burden, irrespective of orthostatic blood pressure changes.

The population of this cohort was in the early mild PD disease stage. The prevalence of abnormal ^123^I-MIBG uptake was comparable to previous studies^7,12,20^. The frequency of OH in early PD patients was similar to another study^21^, and its association with impaired cardiac sympathetic integrity has been reported^4,22^. In this cohort, PD patients with decreased ^123^I-MIBG uptake showed more severe clinical presentations, and they were more susceptible to orthostatic BP drop^7,23^. PD patients with SWEND were associated with orthostatic BP rise, but had less disease burden.

Irrespective of age and disease duration, more severe cardiac sympathetic denervation was related to a larger drop in orthostatic BP (*indirect* component ***a*** → ***b***) and worse disease severity (*direct* path ***a*** → ***c***). Its negative influence was not carried through BP instability to affect PD patients (*indirect* path ***a*** → ***b*** → ***c***). Thus, the *total* effect of sympathetic denervation negatively reflected the global disease burden, and was unaffected by BP lability; more severely impaired cardiac dysautonomia was directly associated with worse clinical outcomes (Table 2). Cardiac denervation inversely paralleled motor, sleep, and autonomic global severity domains in the subcomponent analyses (Table 3).

The inverse association between cardiac sympathetic denervation and worse disease severity could be explained by the degree of accumulated pathology. In a cross-sectional study, PD patients with cardiac sympathetic denervation were speculated to bear more pathologic burden that resulted in lack of compensatory reserve compared to patients with less cardiac sympathetic denervation, thus worse clinical manifestations^7^. This phenotype was further researched in a longitudinal study, and these patients were shown to be at a higher risk of developing motor complications^12^. This temporal association between PD with abnormal ^123^I-MIBG uptake and wearing-off phenomenon implied that pathologic burden at the periphery could mirror central pathophysiologic disease progression as cardiac sympathetic denervation progressed over time^2,4,10,12^. This centripetal degeneration of cardiac sympathetic response was suggested to represent common degenerative process in PD patients^1^, and suggests multiple origination sites for Lewy pathology spread^24^.

The lack of contribution of blood pressure to clinical burdens amid the paths could suggest myocardial ^123^I-MIBG scintigraphy is a potential biomarker that reflects Lewy body pathology^2,17^, in parallel with disease severity. The significant negative association (*indirect* component ***a*** → ***b)***, controlled by age and disease duration, confirmed the contributing action of cardiac sympathetic denervation on OH^4^.

Braak’s schema was disputed because its model failed to explain all the subtypes of PD, especially cardiac denervation in early PD^24^. Recent hypothesis of “body-first” subtype PD incorporates the cardiac pathobiology which may be the answer to the observed differences between normal and abnormal ^123^I-MIBG groups in this study^25,26^. This further enhances the extracranial biomarker role of cardiac denervation in PD.

Early H/M ratio and washout rate which reflect density of the presynaptic cardiac sympathetic nerve endings^2^, its tone^27^, and damaged or failing myocardium^28^, respectively, also manifested similar results that reinforced the hypothesis that ^123^I-MIBG scintigraphy is a mirror of disease burden (Supplementary Table 1, Supplementary Fig. 1).

OHT has seldom been investigated in PD patients, and its characteristics are largely unknown. In this study, despite a difference in estimation of ΔBP during head tilt, the prevalence of OH and OHT were comparable. Interestingly, although the analysis did not gain statistical significance, orthostatic BP incremental change was suggested to be related to worse clinical outcomes. Orthostatic BP drop also displayed a trend for worse outcomes, particularly with respect to ΔSBP. OHT is associated with other types of blood pressure lability, and with cardiovascular risk^18^. In accord with our observation, disrupted blood pressure control could be deleterious, regardless of the directionality of the BP changes^19^.

OHT was significantly associated with normal ^123^I-MIBG that included higher values of delayed H/M ratios. It also displayed lower and negative washout rate, which apparently signified that delayed H/M ratio was greater than early H/M ratio. In this regard, delayed H/M and WR could denote the same as WR contains the other in its equation. This was in correspondence to other studies indicating that delayed H/M and washout rate represented sympathetic tone^2,27^. Its greater sympathetic tone might have allowed normal ^123^I-MIBG group to culminate into higher orthostatic BP rise, particularly SBP, than abnormal ^123^I-MIBG group.

This result suggested that OHT could originate by a different cardiovascular dysregulation mechanism since it was related to normal ^123^I-MIBG. As OH in PD stems from cardiac, extra-cardiac denervation and arterial baroreflex failure^4^, orthostatic ∆BP rise could be the product of compensation of extant cardiac sympathetic tone amid other dysfunctions, which, when lost, could lead to OH. This may imply that OHT could be a prelude to OH in some PD. Our data did not depict any significant difference of washout rate between non-OHT and OHT among preserved ^123^I-MIBG group. Had this been manifested, the sick-but-not-dead phenomenon could have played a role in its pathophysiology;^29^ thus, further support the sequential conversion of OHT into OH as the disease progresses. The nature of OHT in PD needs to be further assessed.

There are several limitations in this study. First, because the enrolled PD patients were in the early phase of the disease, we could not completely exclude the possibility that some had atypical Parkinsonism. In particular, the PD with SWEND patient group might have inadvertently included some multiple system atrophy patients^2^. We attempted to reduce selection bias by using strict clinical diagnostic criteria for PD^30,31^ and structural neuroimaging. Nevertheless, it can be difficult to differentiate PD from multiple system atrophy from a nosological perspective. Second, we also could not exclude patients who had genetic Parkinsonism. Patients with the *Parkin* mutation might not present with nonmotor manifestations and abnormal ^123^I-MIBG uptake^32,33^. This study did not include familial PD or patients with young-onset PD (≤40 years). Third, direct biomarkers that provide objective measurements to assess PD disease severity are not available at the present; in addition, currently useful biomarkers do not always represent clinical symptoms. A clinical assessment-based approach, as in this study, is an alternative that evaluates disease severity, relevant to clinical practice. This study incorporated validated clinical tools that encompassed comprehensive motor and nonmotor disease domains. Fourth, there was not sufficient evidence that ^123^I-MIBG uptake represents myocardial synuclein deposition. Future studies that correlate ^123^I-MIBG myocardial scans with a direct synuclein accumulation biomarker are warranted. Moreover, cardiac imaging with ^18^F-fluorodopa, which may be a better substance to evaluate cardiac sympathetic innervation, was also investigated in PD^16,34^. Diverse studies with cross-validation between different imaging modalities will strongly enhance the results of this research. Finally, because early patients with mild PD stage were enrolled, the scores of questionnaires tended to center at the left of the scale with relatively large standard deviation. A larger recruitment of PD by engagement of multi-clinics to reduce the bias is warranted.

In this cohort, cardiac sympathetic denervation negatively predicted disease severity, independently of orthostatic blood pressure changes. Even though cardiac denervation is a contributor to orthostatic hypotension, the effect of cardiac denervation on disease burdens was not mediated by orthostatic blood pressure changes. Cardiac sympathetic denervation was suggested to represent the disease burden, in parallel with pathologic severity. Orthostatic hypertension was also observed, and it could potentially contribute to PD symptom clinical severity.

## Methods

### Patients

This study was approved by the Institutional Review Board of Seoul St. Mary’s Hospital, and all subjects provided written informed consent to participate. All experiments were conducted in accordance with relevant guidelines and regulations. The study was registered (Identification Number: KCT0005552) in the Clinical Research Information Service (CRIS; http://cris.nih.go.kr), which is an online clinical trial registration system established by the Korea Centers for Disease Control and Prevention (KCDC) with support from the Korea Ministry of Health and Welfare (KMOHW) and is affiliated with the Primary Registries in the World Health Organization (WHO) Registry Network.

Two hundreds twenty-seven de novo and drug-naïve PD patients between April 2014 and January 2020 were enrolled. PD was diagnosed based on the UK Parkinson’s Disease Society Brain Bank^30^, and its diagnosis was supported by positron emission tomography imaging studies using ^18^F-N-(3-fluoropropyl)-2beta-carbon ethoxy-3beta-(4-iodophenyl) nortropane^31^. All patients had decreased dopamine transporter uptake in the striatum, mainly in the posterior putamen. Patients underwent brain magnetic resonance imaging (MRI) to exclude secondary causes for this finding.

Demographics such as age, sex, body mass index, disease duration, smoking status and history of hypertension, diabetes mellitus, and dyslipidemia were investigated. Disease severity was investigated with the UPDRS and H&Y stage. Global cognition was assessed by the MMSE and CDR.

Patients were disqualified from the study if they had any of the following indications: (1) any symptoms or signs of atypical and/or secondary Parkinsonism, (2) positive family history of Parkinsonism by pedigree analysis, which included first degree relatives, (3) documentation of atrial fibrillation during the head up tilt test, (4) history of diabetic neuropathy, (5) history of symptomatic stroke that could affect general cognition and performance, and (6) history of medications such as tricyclic antidepressants or benzodiazepines that influence autonomic functions or patients who were taking medications at the time of diagnosis known to influence the central dopaminergic, noradrenergic, and/or serotonergic systems.

Patients were followed every 2–6 months for a minimum of 12 months from the time they began taking dopaminergic medication, and their diagnosis was reaffirmed after at least 12 months of follow-up by two neurologists (S.-W.Y., J.-S.K.).

### Questionnaires

The patients were evaluated with the following questionnaires: (1) NMSS^35^, (2) MADRS^36^, (3) ESS^37^, (4) PDSS-2^38^, (5) RBDSQ^39^, (6) OHQ^40^, and (7) the SCOPA-AUT^41^. Part I and II of the OHQ were summed separately. Because the SCOPA-AUT sexual dysfunction subsection had too many missing values, this subsection was omitted from that questionnaire’s summation. Missing values of sexual subsection was due to patients’ reluctance to reveal their sexual activities to the examiner. The sums of each questionnaire were analyzed.

### Composite scores

Motor severity (motor composite score) was gauged by averaging the z-scores of the scaled UPDRS II and UPDRS III. The sums of each individual questionnaire for nonmotor features were standardized to z-scores. Nonmotor features were also divided into the sleep domain (sleep composite score: average z-scores for the ESS, PDSS-2, RBDSQ), and the autonomic domain (autonomic composite score: average z-scores for the OHQ and SCOPA-AUT), and were further analyzed separately. The affection domain was not analyzed because the MADRS data were not normally distributed, and the NMSS was excluded from subcomponent analysis due to its inclusive psychometric properties.

Parkinson’s disease overall burden (the global composite score) was estimated by averaging the scaled z-scores of all motor and nonmotor assessments. Higher z-scores indicated worse severity. All questionnaires were evaluated by investigators blind to patient clinical information.

### Head-up tilt test

All patients were tested in the full resting state. Continuous electrocardiograph leads and non-invasive BP monitoring equipment were applied to the patients (YM6000, Mediana Tech, Redmond, WA, USA). A supine position was maintained for 20 min during recording of BPs and heart rates every 5 min, before tilting to 60 degrees (ENRAF NONIUS, Rotterdam, The Netherlands). At the tilted position, the same measurements were taken at 0, 3, 5, 10, 15, and 20 min.

After excluding the first supine BP at 0 min, average supine systolic and diastolic BPs were estimated from the measurements at 5, 10, 15, and 20 min. SH was defined if the average supine systolic and/or diastolic BPs (SBP/DBP) were ≥140/90 mmHg^42^.

The lowest SBP/DBP at 3 or 5 minutes during the tilted position were selected for the diagnosis of OH. The orthostatic BP changes in systole (*Δ*SBP_min_) and diastole (*Δ*DBP_min_) were also calculated (supine average BP minus *lowest* orthostatic BP). When patients were hypertensive with ≥140/90 mmHg in the supine position, *Δ*SBP_min_ and/or *Δ*DBP_min_ ≥30/15 mmHg within 5 min was applied to define OH; otherwise, *Δ*SBP_min_ and/or *Δ*DBP_min_ ≥20/10 mmHg were adopted^43^.

The highest SBP/DBP among tilted measurements at 3, 5, 10, 15, and 20 min were re-selected, and the orthostatic ∆BPs were calculated from the average BPs (supine average BP minus *highest* orthostatic BP). PD patients with SH were defined as having orthostatic hypertension (OHT) if *Δ*SBP_max_ and/or *Δ*DBP_max_ was ≤ −20/10 mmHg. PD patients without SH were categorized as OHT when their orthostatic BP_max_ was ≥140/90 mmHg or *Δ*BP_max_ was ≤ −20/10 mmHg^19^.

Positive orthostatic *Δ*BP indicated a drop in standing BP, and the negative *Δ*BP an increase in standing BP.

### ^123^I-metaiodobenzylguanidine (^123^I-MIBG) scintigraphy

^123^I-MIBG scintigraphy was performed using a dual-head camera equipped with a low-energy, high-resolution collimator (Siemens), and data were collected at 30-min (early) and 2-h (delayed) time points after the injection of 111 MBq of ^123^I-MIBG. A static image was obtained with a 128 × 128 matrix. Regions of interest were manually drawn around the heart and mediastinum. Tracer uptake was measured within each region of interest to calculate the H/M ratio. The lower limit of the reference value for delayed H/M ratio was calculated to be 1.78^12,13^. A delayed H/M ratio < 1.78 was defined as abnormal. Washout rate was calculated as the following: [(early H/M ratio − late H/M ratio)/early H/M ratio] x 100^13^.

### Statistical analyses

All statistical analyses were conducted with Jamovi software (version 1.6; retrieved from https://www.jamovi.org) for the Mac, a graphical user interface for R, with the additional jAMM module. The jAMM module provides a GLM mediation model that utilizes the *lavaan* R package^44^. Descriptive statistics, and independent or Welch’s t-tests, the Mann–Whitney *U* test or Fisher’s exact test were performed as appropriate. Mediation models, partialized by the covariates of age and disease duration, were manipulated to assess whether there was a mediating role for orthostatic ∆BP_min_ (***b***) between cardiac sympathetic denervation (***a***) and disease burden (***c***). The *direct* association between delayed H/M ratio and composite scores (***a*** → ***c***) was established within the context of an *indirect* effect of orthostatic ∆BP_min_ (***a*** → ***b*** → ***c***). Their relative roles were estimated using the maximum likelihood method^45^ and statistical significance was defined as a two-tailed *p* value < 0.05.

### Reporting summary

Further information on research design is available in the Nature Research Reporting Summary linked to this article.

## Supplementary information

Supplementary Information

Reporting Summary

## Data Availability

Anonymized data generated during the current study are available from the corresponding author on reasonable request from individuals affiliated with research or health care institutions.

## Code availability

Jamovi is a statistical spreadsheet and graphical user interface (GUI) for R. All the analyses were performed basically using jamovi software as mentioned in the method, statistical analysis section. The R package mentioned was utilized within jamovi GUI.
