# Supplementary material for: Cardiac sympathetic burden reflects Parkinson disease burden, regardless of high or low orthostatic blood pressure changes
Source: NPJ Parkinsons Dis. 2021 Aug 12;7:71. doi: 10.1038/s41531-021-00217-3 (PMC8361133; doi:10.1038/s41531-021-00217-3)

Supplementary table 1. The mediating effects of orthostatic blood pressure changes (ΔSBP_{min}, ΔDBP_{min}) for different predictors

Predictor: Early H/M ratio

Mediator (*b*): ΔSBP_{min}								
Type	Effect	Estimate	SE	Lower 95% CI	Upper 95% CI	β	z	p
Indirect	*a' ⟶ b ⟶ c*	-0.036	0.046	-0.136	0.057	-0.016	-0.777	0.437
Component	*a' ⟶ b*	-13.276	2.687	-18.570	-7.805	-0.307	-4.940	< 0.001
	b ⟶ c	0.003	0.003	-0.004	0.010	0.051	0.791	0.429
Direct	*a' ⟶ c*	-0.477	0.151	-0.781	-0.169	-0.207	-3.168	0.001
Total	Indirect + direct	-0.510	0.147	-0.798	-0.222	-0.221	-3.470	< 0.001
Mediator (*b*): ΔDBP_{min}								
Type	Effect	Estimate	SE	Lower 95% CI	Upper 95% CI	β	z	p
Indirect	*a' ⟶ b ⟶ c*	0.007	0.035	-0.054	0.081	0.003	0.216	0.829
Component	*a' ⟶ b*	-6.128	1.566	-8.969	-3.064	-0.242	-3.913	< 0.001
	b ⟶ c	-0.001	0.006	-0.013	0.010	-0.013	-0.221	0.825
Direct	*a' ⟶ c*	-0.517	0.141	-0.811	-0.256	-0.224	-3.666	< 0.001
Total	Indirect + direct	-0.510	0.147	-0.798	-0.222	-0.221	-3.470	< 0.001

Predictor: Washout rate

Mediator (*b*): ΔSBP_{min}								
Type	Effect	Estimate	SE	Lower 95% CI	Upper 95% CI	β	z	p
Indirect	*a" ⟶ b ⟶ c*	0.001	0.001	-0.123	0.004	0.016	1.174	0.241
Component	*a" ⟶ b*	0.320	0.094	0.131	0.506	0.202	3.410	0.001
	b ⟶ c	0.004	0.003	-0.002	0.011	0.078	1.299	0.194
Direct	*a" ⟶ c*	0.014	0.005	0.006	0.025	0.171	2.922	0.003
Total	Indirect + direct	0.016	0.005	0.005	0.026	0.184	2.896	0.004
Mediator (*b*): ΔDBP_{min}								
Type	Effect	Estimate	SE	Lower 95% CI	Upper 95% CI	β	z	p
Indirect	*a" ⟶ b ⟶ c*	0.029	0.136	-0.001	0.002	0.002	0.214	0.830
Component	*a" ⟶ b*	0.125	0.058	0.007	0.241	0.134	2.140	0.032
	b ⟶ c	0.001	0.006	-0.009	0.013	0.014	0.230	0.818
Direct	*a" ⟶ c*	0.016	0.005	0.005	0.026	0.183	3.026	0.002
Total	Indirect + direct	0.016	0.005	0.005	0.026	0.184	2.896	0.004

SBP, systolic blood pressure; *DBP*, diastolic blood pressure; *SE*, standard error
a': Early heart-to-mediastinum (H/M) ratio; *a"*: Washout rate; *b*: ΔSBP_{min} or ΔDBP_{min}; *c*: global composite score

The mediation model was controlled by age and disease duration. Multicollinearity was prevented by m ean centering method.
Confidence intervals (CIs) were calculated with percentile bootstrap (n=1,000) method. Betas are completely standardized effect sizes.

Supplementary figure 1. Path diagram of mediation analysis for different predictors

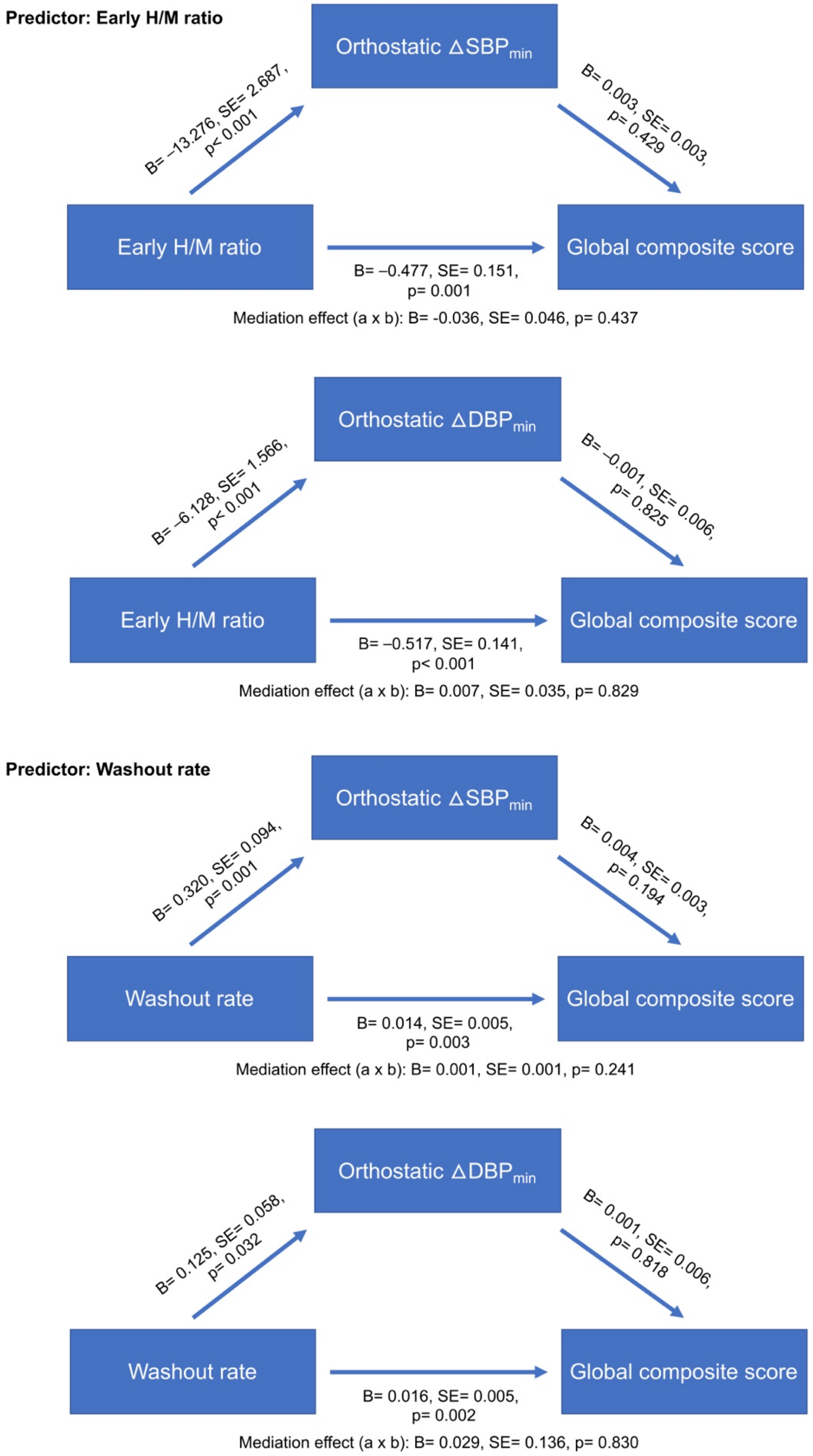

Supplementary figure 2. Bidirectionality of orthostatic ΔBP_{max}

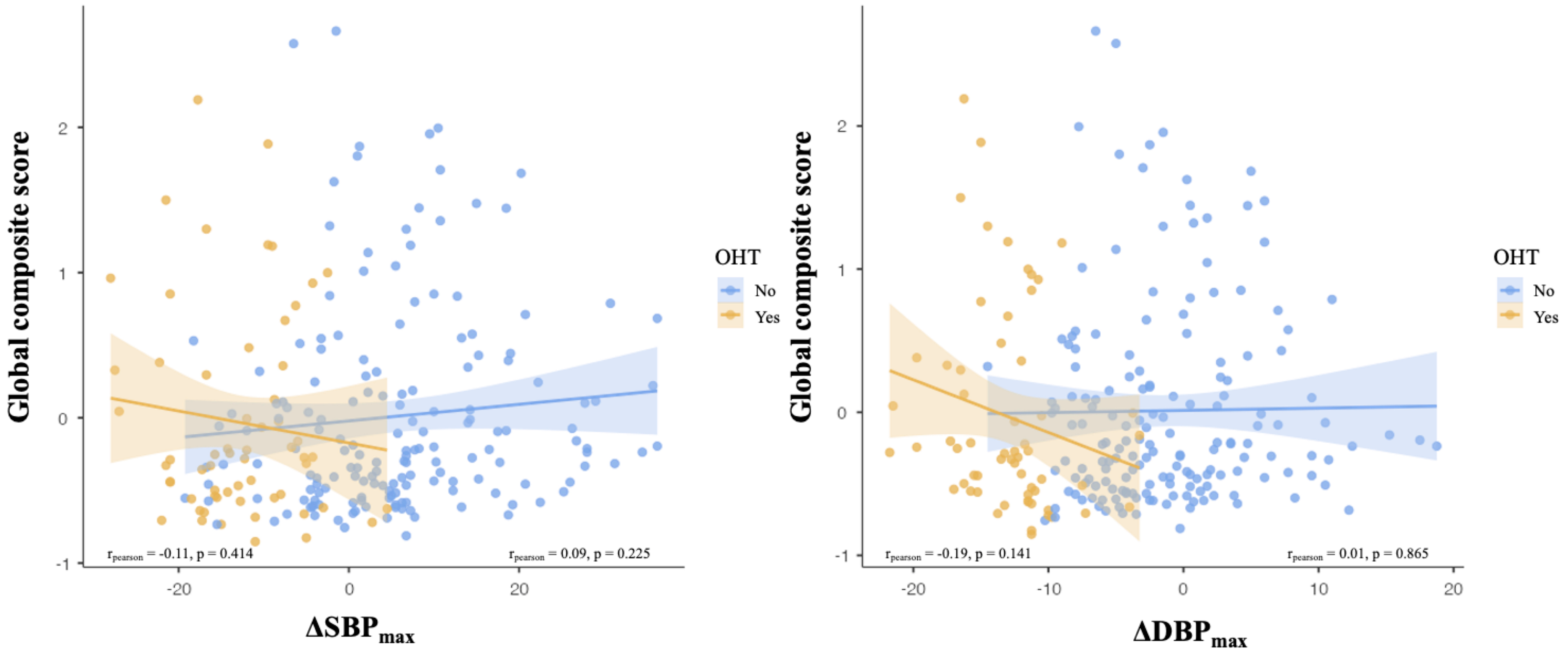

Supplement: Supplementary file 1 — Supplementary Information [file 41531_2021_217_MOESM1_ESM.pdf]
