# Supplementary material for: Cardiac sympathetic burden reflects Parkinson disease burden, regardless of high or low orthostatic blood pressure changes
Source: NPJ Parkinsons Dis. 2021 Aug 12;7:71. doi: 10.1038/s41531-021-00217-3 (PMC8361133; doi:10.1038/s41531-021-00217-3)
Supplement: Supplementary file 2 — Reporting Summary [file 41531_2021_217_MOESM2_ESM.pdf]

nature research

Corresponding author(s): Joong-Seok Kim

Last updated by author(s): Jul 13, 2021

Reporting Summary

Nature Research wishes to improve the reproducibility of the work that we publish. This form provides structure for consistency and transparency in reporting. For further information on Nature Research policies, see our Editorial Policies and the Editorial Policy Checklist.

Statistics

For all statistical analyses, confirm that the following items are present in the figure legend, table legend, main text, or Methods section.

n/a	Confirmed	
☐	☒	The exact sample size (*n*) for each experimental group/condition, given as a discrete number and unit of measurement
☐	☒	A statement on whether measurements were taken from distinct samples or whether the same sample was measured repeatedly
☐	☒	The statistical test(s) used AND whether they are one- or two-sided<br>*Only common tests should be described solely by name; describe more complex techniques in the Methods section.*
☐	☒	A description of all covariates tested
☐	☒	A description of any assumptions or corrections, such as tests of normality and adjustment for multiple comparisons
☐	☒	A full description of the statistical parameters including central tendency (e.g. means) or other basic estimates (e.g. regression coefficient) AND variation (e.g. standard deviation) or associated estimates of uncertainty (e.g. confidence intervals)
☐	☒	For null hypothesis testing, the test statistic (e.g. *F*, *t*, *r*) with confidence intervals, effect sizes, degrees of freedom and *P* value noted<br>*Give P values as exact values whenever suitable.*
☒	☐	For Bayesian analysis, information on the choice of priors and Markov chain Monte Carlo settings
☒	☐	For hierarchical and complex designs, identification of the appropriate level for tests and full reporting of outcomes
☒	☐	Estimates of effect sizes (e.g. Cohen's *d*, Pearson's *r*), indicating how they were calculated

Our web collection on statistics for biologists contains articles on many of the points above.

Software and code

Policy information about availability of computer code

Data collection	Data collection was done by using Microsoft Excel. The files were then converted into csv files for statistical programs.
Data analysis	For data analysis, jamovi software (version 1.6) and R software (version 3.6) with the additional jAMM module were used. The jAMM module provides a GLM mediation model that utilizes the lavaan R package.

For manuscripts utilizing custom algorithms or software that are central to the research but not yet described in published literature, software must be made available to editors and reviewers. We strongly encourage code deposition in a community repository (e.g. GitHub). See the Nature Research guidelines for submitting code & software for further information.

Data

Policy information about availability of data

All manuscripts must include a data availability statement. This statement should provide the following information, where applicable:

- Accession codes, unique identifiers, or web links for publicly available datasets
- A list of figures that have associated raw data
- A description of any restrictions on data availability

Anonymized data generated during the current study are available from the corresponding author on reasonable request from individuals affiliated with research or health care institutions.

Field-specific reporting

Please select the one below that is the best fit for your research. If you are not sure, read the appropriate sections before making your selection.

☒ Life sciences ☐ Behavioural & social sciences ☐ Ecological, evolutionary & environmental sciences

For a reference copy of the document with all sections, see nature.com/documents/nr-reporting-summary-flat.pdf

Life sciences study design

All studies must disclose on these points even when the disclosure is negative.

Sample size	Sample size was not calculated in this cross-sectional and observational study. The enrolled number of participants were part of Parkinson's disease cohort study of our institution. The authors tried to recruit at least thirty patients for each group to guarantee normality, thus at least 100 PD patients in total.
Data exclusions	Among the subjects who were initially enrolled, those with missing values on each test were excluded.
Replication	The results of this study was reproducible. The tests were re-checked with above mentioned statistical software (jamovi, R).
Randomization	As this study is exploratory, randomization was not performed. The mediation models were adjusted by covariates in this study, when appropriate. These covariates are generally controlled in data analyses of this kind. They are well-known to influence the clinical outcomes.
Blinding	Blinding was not relevant in this study because of its design, an observatory cohort.

Reporting for specific materials, systems and methods

We require information from authors about some types of materials, experimental systems and methods used in many studies. Here, indicate whether each material, system or method listed is relevant to your study. If you are not sure if a list item applies to your research, read the appropriate section before selecting a response.

Materials & experimental systems

n/a	Involved in the study	
☒	☐	Antibodies
☒	☐	Eukaryotic cell lines
☒	☐	Palaeontology and archaeology
☒	☐	Animals and other organisms
☐	☒	Human research participants
☒	☐	Clinical data
☒	☐	Dual use research of concern

Methods

n/a	Involved in the study	
☒	☐	ChIP-seq
☒	☐	Flow cytometry
☒	☐	MRI-based neuroimaging

Human research participants

Policy information about studies involving human research participants

Population characteristics	Research sample is selected among patients who visit our institution's movement clinic at Seoul St Mary's hospital. The mean age was 69.6 ± 9.2 years old and 105 (46.3%) were female. The sample is representative of PD population of a single center. It is from our cohort registry that collects data retrospectively and prospectively. Participants with analyzable data were selected from this registry, so the sample size was not calculated.
Recruitment	Participants who visited our movement clinic were recruited with their consent. As this study enrolled at a single center, this may cause selection bias.
Ethics oversight	This study was approved by the Institutional Review Board at Seoul St. Mary's Hospital.

Note that full information on the approval of the study protocol must also be provided in the manuscript.
